# Health workforce needs and health policy responses to COVID-19: a European comparative assessment: Ellen Kuhlmann

**DOI:** 10.1093/eurpub/ckac129.042

**Published:** 2022-10-25

**Authors:** E Kuhlmann, M-G Brînzac, V Burau, T Correia, M Falkenbach, M-I Ungureanu

**Affiliations:** 1 Clinic for Rheumatology and Immunology, Hannover Medical School, Hannover, Germany; 2 Faculty of Political, Administrative and Communication Sciences, Babeș-Bolyai University, Cluj-Napoca, Romania; 3 Department of Political Science, University of Aarhus, Aarhus, Denmark; 4 Department of Public Health, University of Aarhus, Aarhus, Denmark; 5 Instituto de Higiene e Medicina Tropical, Universidade Nova de Lisboa, Lisbon, Portugal; 6 Department of Public and Ecosystem Health, Cornell University, Ithaca, New York, USA

## Abstract

**Background:**

The COVID-19 pandemic revealed the importance of the health workforce for health system resilience. This study aims to explore whether and how healthcare system in Europe have responded to new emergent needs and transformed their health workforce policies.

**Methods:**

A qualitative comparative approach is applied, based on multi-level governance theory and a rapid assessment of three areas of health workforce policy: mental health, gender equality, and public health competencies. We consider two years of the pandemic with a focus on recent waves, October 2021-January 2022. Denmark, Germany, Portugal, Romania and Switzerland are selected for comparison, representing different health systems, health workforce conditions and COVID-19 indicators in the European Union and European Economic Area.

**Results:**

Across countries the pandemic has highlighted mental health needs, persisting gender inequalities and demand for public health competencies. Our comparison reveals similar weaknesses and governance gaps. (1) Mental health needs of healthcare workers are increasingly recognised (more strongly in Denmark and less in Romania with the other countries clustering in-between); however, health workers’ perceptions are not used as guidance and effective programmes are lacking. (2) The situation is worst in relation to gender equality goals that are largely ignored in pandemic policy and recovery plans. (3) Public health competences are more advanced and integrated in the NHS systems in Denmark and Portugal, but no country has taken action to innovate health workforce education and strengthen public health.

**Conclusions:**

The comparative assessment highlights that health systems failed to adequately respond to health workforce needs and the COVID-19 challenges. Action has to be taken to implement participatory governance and step up efforts towards more responsive and resilient health workforce policy.

